# Movement-Related Cortical Potentials in Embodied Virtual Mirror Visual Feedback

**DOI:** 10.3389/fneur.2021.646886

**Published:** 2021-06-15

**Authors:** Gangadhar Garipelli, Tamara Rossy, Daniel Perez-Marcos, Jane Jöhr, Karin Diserens

**Affiliations:** ^1^MindMaze SA, Lausanne, Switzerland; ^2^School of Life Sciences, Ecole Polytechnique Fédérale de Lausanne, Lausanne, Switzerland; ^3^Acute Neurorehabilitation Unit, Department of Clinical Neurosciences, Centre Hospitalier Universitaire Vaudois, Lausanne, Switzerland

**Keywords:** stroke, neurorehabilitation, virtual reality, electroencephalogram, cortical excitability, movement-related cortical potentials, mirror therapy, mirror visual feedback

## Abstract

**Background:** Mirror therapy is thought to drive interhemispheric communication, resulting in a balanced activation. We hypothesized that embodied virtual mirror visual feedback (VR-MVF) presented on a computer screen may produce a similar activation. In this proof-of-concept study, we investigated differences in movement-related cortical potentials (MRCPs) in the electroencephalogram (EEG) from different visual feedback of user movements in 1 stroke patient and 13 age-matched adults.

**Methods:** A 60-year-old right-handed (Edinburgh score >95) male ischemic stroke [left paramedian pontine, National Institutes of Health Stroke Scale (NIHSS) = 6] patient and 13 age-matched right-handed (Edinburgh score >80) healthy adults (58 ± 9 years; six female) participated in the study. We recorded 16-electrode electroencephalogram (EEG), while participants performed planar center-out movements in two embodied visual feedback conditions: (i) direct (movements translated to the avatar's ipsilateral side) and (ii) mirror (movements translated to the avatar's contralateral side) with left (*direct left*/*mirror left*) or right (*direct right/mirror right*) arms.

**Results:** As hypothesized, we observed more balanced MRCP hemispheric negativity in the *mirror right* compared to the *direct right* condition [statistically significant at the FC4 electrode; 99.9% CI, (0.81, 13)]. MRCPs in the stroke participant showed reduced lateralized negativity in the *direct left* (non-paretic) situation compared to healthy participants. Interestingly, the potentials were stronger in the *mirror left* (non-paretic) compared to *direct left* case, with significantly more bilateral negativity at FC3 [95% CI (0.758 13.2)] and C2 [95% CI (0.04 9.52)].

**Conclusions:** Embodied mirror visual feedback is likely to influence bilateral sensorimotor cortical subthreshold activity during movement preparation and execution observed in MRCPs in both healthy participants and a stroke patient.

## Introduction

Post-stroke motor rehabilitation techniques, such as constraint-induced movement therapy (1), require residual movements of the affected limb. Treatments involving mirror therapy (MT) (2, 3), motor imagery (4), and action observation therapy (5) have recently emerged as alternatives for severely disabled patients. In MT, the mirror visual feedback (MVF) is typically delivered using a mirror placed in the parasagittal plane of the body (either upper or lower) that reflects the unaffected limb movements in the position of the other limb, hence resulting in a perception of movement of the affected limb. MT has been suggested to drive interhemispheric communication leading to a more balanced cortical activation (2). A recent review found that MVF could exert a strong influence on the motor network, mainly through increased cognitive load in action control (6). MVF was found to enhance the excitability of the ipsilateral primary motor cortex that projects to the inactive hand/arm, as described in some neurophysiological studies [increased motor-evoked potentials (7) and blood–oxygen-level-dependent (BOLD) activity (8); changes in lateralization of readiness potentials in electroencephalogram (EEG) (9); and beta band activity in magnetoencephalogram (MEG) (10)]. Movement planning and execution are associated with slow negative potentials at the central electrodes in scalp EEG (11), which is thought to reflect the subthreshold activity of the neural tissue and hence the regulation of cortical excitability. These potentials are found to be sensitive to brain damage (11–14). Interestingly, recent studies showed that these potentials could be manipulated using MVF in healthy participants (9, 15) and stroke patients (15).

Virtual reality (VR) applications are emerging in post-stroke motor rehabilitation procedures (16). VR may offer a controlled medium to integrate evidence-based principles such as the MT (3), into a standard of care, increasing motivation and engagement, which are necessary for delivering higher therapy dose levels and ensuring adherence (17). VR-mediated embodied mirror visual feedback (VR-MVF) may also engage similar neural circuits as MVF (15). Recently, we have underlined the importance of making neurophysiological measurements for correlating clinical evolution in the very acute phase (18). In the current proof-of-concept study, we investigated changes in the lateralization of movement-related cortical potentials (MRCPs) in EEGs recorded from a stroke participant and 13 age-matched healthy participants performing a reaching task using embodied VR-MVF.

## Materials and Methods

### Participants

The Human Research Ethics Committee of the Canton of Vaud, Switzerland, approved the study. The study was conducted in the Acute Neurorehabilitation Unit of the Centre Hospitalier Universitaire Vaudois (CHUV) in Lausanne, Switzerland. All participants provided written informed consent before the experiment.

#### Healthy Participants

A cohort of 13 healthy right-handed volunteers (mean age, 58.23 ± 9.47 years) was recruited for this study.

#### Stroke Participant

We recruited a 60-year-old right-handed (Edinburgh score >95) male subject who survived an ischemic stroke (left paramedian pontine) of atheromatous origin, which caused hemiparesis of the right side. The participant was hospitalized on day 0, and assessments (*Outcome measures*) were obtained on day 8 (preintervention, T1), day 13 (post-intervention, T2), and day 706 (follow-up T3). The patient performed the VR task on three consecutive days, namely, days 9, 10, and 11, using the MindMotion^TM^ PRO VR system (*Experimental setup*) at the patient's bedside in the acute neurorehabilitation center. The patient also received ~60 min of full-body occupational therapy during the hospital stay.

### Experimental Setup

**Figure 1:**
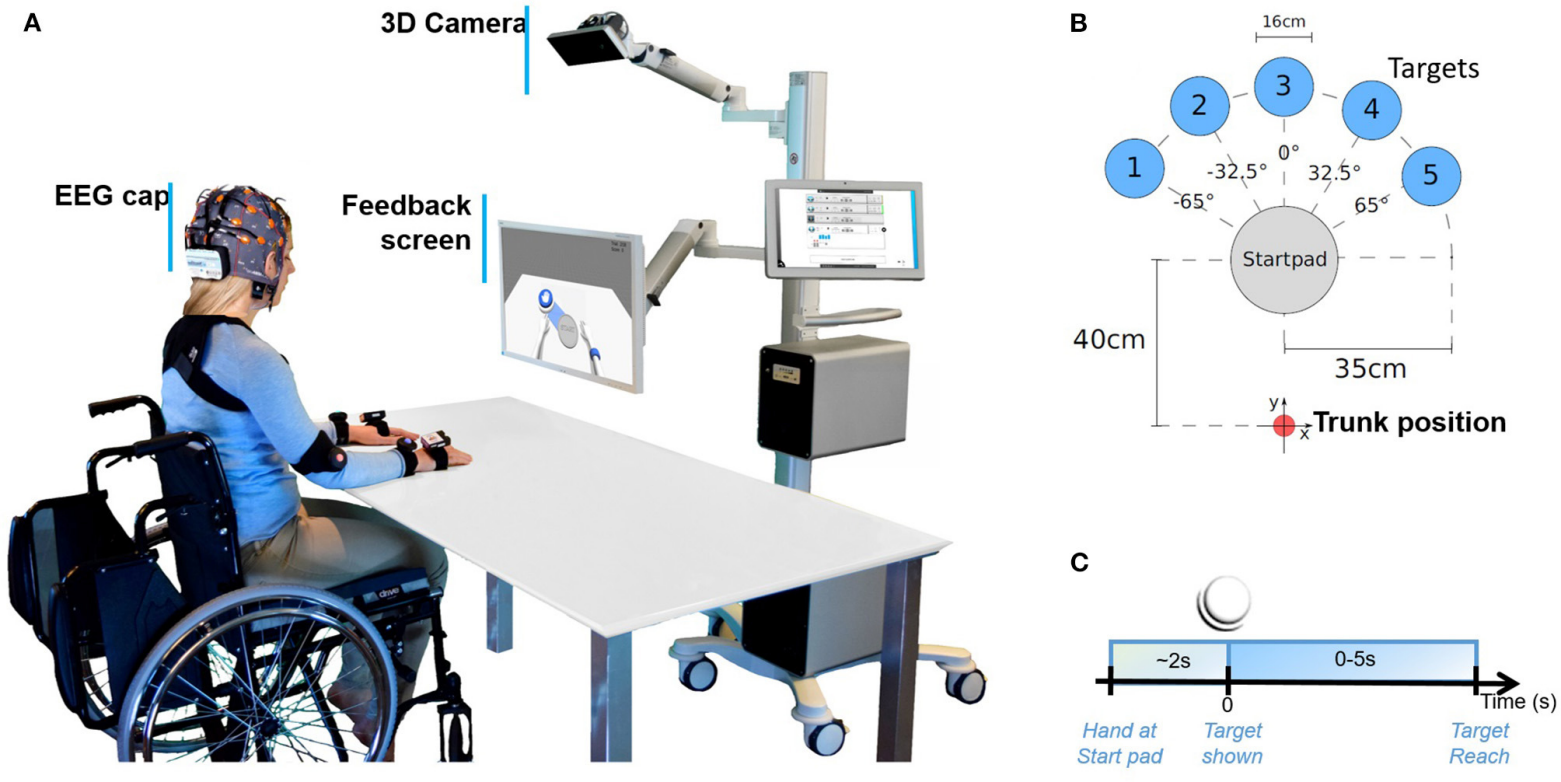
Illustration of the experimental setup. **(A)** A participant performing centered-out movements over the 2D surface of a table in front of the MindMotion^TM^ PRO virtual reality platform. A 3D camera tracks the participant's movements and projects them onto a feedback screen. **(B)** Approximate locations of the five virtual targets are illustrated with respect to the participant's trunk position. **(C)** Timeline of events of each repetition of the center-out reaching task.

Participants were asked to perform upper limb center-out reaching movements in a 2-dimensional plane (over a physical table) to five locations randomly presented using the MindMotion^TM^ PRO (MindMaze SA, Lausanne, Switzerland) VR rehabilitation platform. The participants sat in a comfortable chair or wheelchair in front of a table as shown in Figure 1A. The MindMotion^TM^ PRO system consists of a motion-capture camera (placed at a height of ~180 cm) for tracking the upper extremity. In addition, a touch screen for the therapist to set up the training session and a monitor (placed at ~120 cm from participant) to display the embodied feedback via an avatar that reproduces participants' movements in real time were used. In a typical trial, participants first placed a hand on the Start pad, when ready. After a random time (maximum of 2 s), a target (35 cm away) was exposed at one of the five locations (each 32.5° apart), as described in Figure 1B. Participants were then told to reach toward the target at their own pace along the line linking the Start pad and target object. Approximately 1 s after reaching the target, participants received a score corresponding to the trajectory accuracy. Participants were asked to use their left or right arm to perform the reaching task. The embodied feedback was provided using two mapping modes: (i) Direct, where movements of the active arm were translated to the same side of the avatar or (ii) Mirror mapping, where the movement of the arm is exchanged over the mid-sagittal plane while keeping the shoulder internal/external rotation and forearm pronation and supination the same.

### Paradigms

#### Healthy Participants

**Figure 2:**
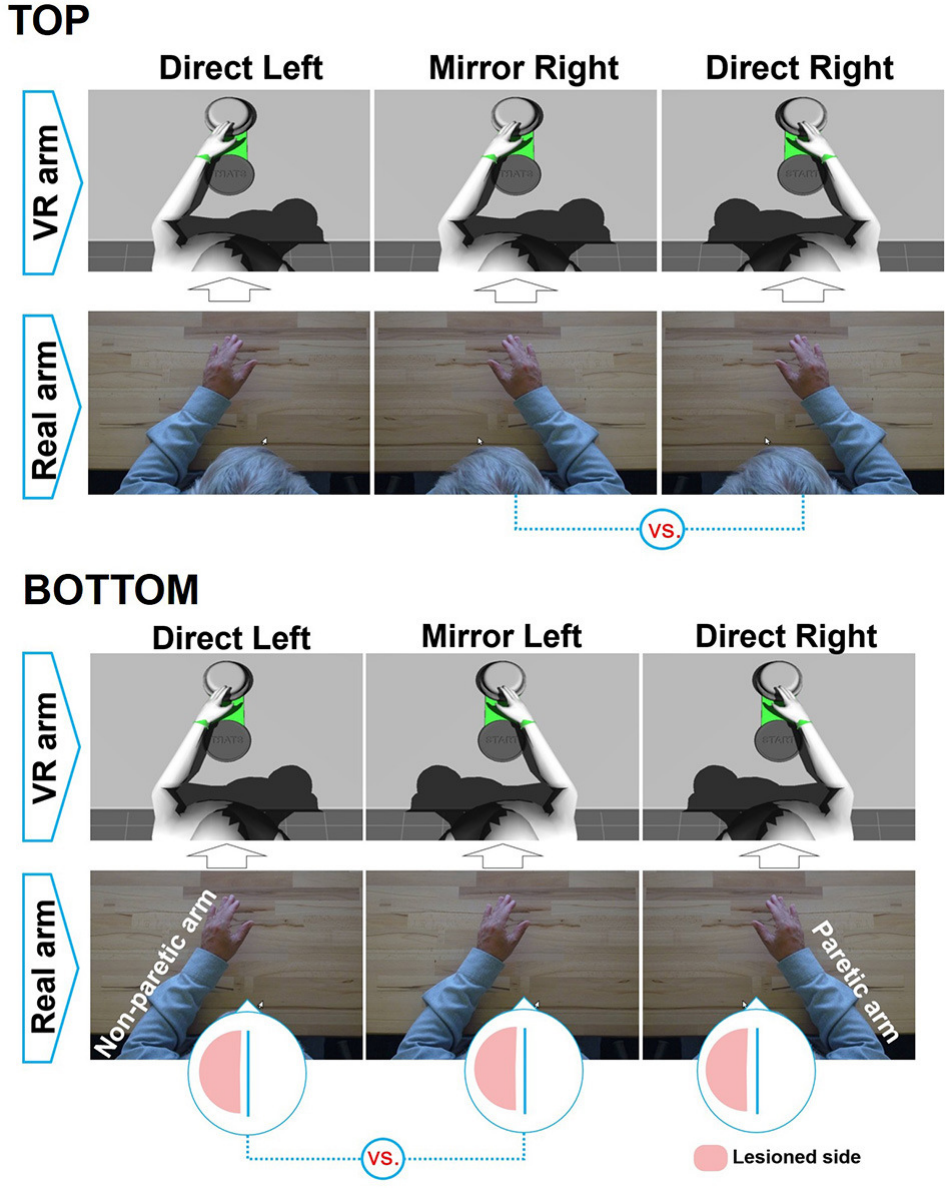
Illustrated feedback conditions **(top)** for healthy participants and **(bottom)** for the stroke participant. The comparisons made to investigate MVF are shown underneath.

Healthy participants performed the planar center-out reaching movements with *direct* or *mirror* feedback in three conditions as described in Figure 2 (top): (a) *direct left*, the participant moves the left arm, and the avatar reproduces the movement with its left arm; (b) *mirror right*, the participant moves the right arm and the avatar reproduces the movement with its left arm; and (c) *direct right*, the participant moves the right arm and the avatar reproduces the movement with its right arm. Each participant performed ~100 repetitions per condition per session with conditions presented in blocks (25 repetitions) of a random order.

#### Stroke Participant

The stroke participant performed the center-out reaching tasks over three consecutive days (Figure 2, bottom), beginning on day 9 in the following three conditions: (a) *direct left* (40, 60, and 110 repetitions on days 9, 10, and 11, respectively); (b) *mirror left*, the participant moves the left arm (non-paretic), and the avatar moves its right arm (40, 95, and 80 repetitions on days 9, 10, and 11, respectively); and (c) *direct right*, the participant tried to use the right arm (paretic) with 10, 85, and 90 repetitions on days 9, 10, and 11, respectively. The order of the conditions was chosen according to the patient's comfort, where we started with direct right condition and shifted to other conditions whenever the patient reported fatigue in the paretic arm, allowing the arm to recover.

### Data Collection

#### Electroencephalogram

We recorded full-band EEG (fbEEG) in 10–20 international system with 16 electrodes (FC3, FCz, FC4, C5, C3, C1, Cz, C2, C4, C6, CP3, CP1, CPz, CP2, CP4, and Pz) using the *g.USBamp* recording system (ground at AFz electrode; right earlobe reference) for all participants. The EEG data was sampled at 512 Hz and synchronized with MindMotion^TM^ PRO exercise events that were detected using a 3D motion-capture system.

#### Outcome Measures

We measured the patient's stroke severity using the National Institutes of Health Stroke Scale (NIHSS), motor impairment using the Fugl–Meyer Assessment for Upper Extremity subscale (FMA-UE) (19), and motor function using the Frenchay Arm Test (FAT) (20) at T1, T2, and T3 (*Stroke participant*).

### Data Processing

We performed data processing using Matlab R2013a (Mathworks Inc., Natick, MA, USA).

#### Electroencephalogram

First, the DC offset of the EEG recordings was reduced, removing the first sample from each electrode data. Then, data underwent downsampling from 512 to 64 Hz (zero-phase low-pass third-order Butterworth filter; *f*_*c*_ = 25.6 Hz). After this, the data were band-pass filtered in the range of 0.1–1.5 Hz corresponding to the spectral content of slow cortical potentials (SCPs) (21). For healthy participants, data were re-referenced to Pz electrode activity [MRCPs at the Pz electrode are expected to be small or zero, as the electrode is orthogonally located with respect to its source at the near-central electrodes (11)]. For the stroke participant's recordings, the data were re-referenced with respect to the average activity of the C5 and C6 electrodes (an approximate average of the earlobe reference).

#### Electroencephalogram Grand Averages

Trials were extracted using a −2 to +2 s time window around movement onset at zero seconds (Figure 1C). For each trial, we detected movement onset using the 3D motion-capture system as the moment when the avatar's hand left the Start pad in the VR. We rejected trials with too-late movement onset or multiple movement onsets. The remaining trials were then corrected using a baseline activity sample at −1.5 s. Trials that exceeded potentials of ±120 μV at any electrode were discarded from the analysis. The remaining trial data were averaged across all healthy participants for each condition and electrode separately. The stroke participant's EEG epoch data were treated using the same processing steps. However, since only a small number of trials remained for the patient in *direct right* (paretic arm) after artifact rejection, we limited further analysis to *direct left* and *mirror left* conditions. We produced topographic plots of the average activity at each channel over a 25-ms time window, selected using the time point that corresponded to the maximal difference between the observed conditions.

#### Statistical Analysis

For healthy participant data, we used ANOVA to assess differences in the negativity of MRCPs between electrodes depending on their location. We used a single, three-leveled factor: *direct left, direct right*, or *mirror right* conditions. The observations consisted of the pooled data for all participants using the mean activity corresponding to the same temporal window as the topographic map described in *Electroencephalogram grand averages*. We then used Tukey's test for pairwise differences in *direct right* vs. *direct left* and *direct right* vs. *mirror right*. Similarly, for the stroke participant's data, we performed Tukey's test for each electrode separately to assess the differences between *direct left (non-paretic)* and *mirror left (non-paretic)* conditions.

## Results

### Healthy Participants

**Figure 3:**
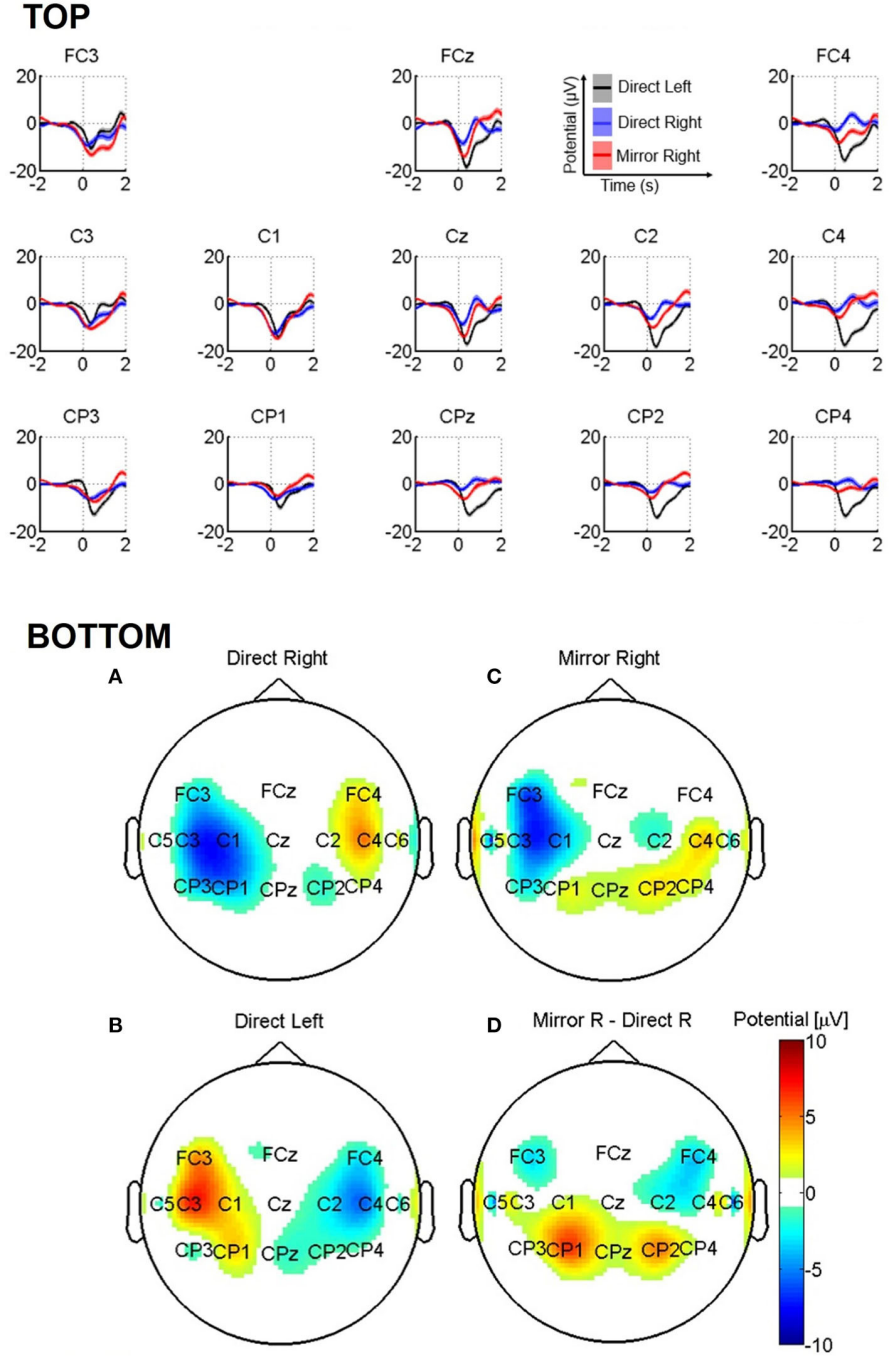
**(Top)** Grand-average MRCPs of healthy participants for *direct left, direct right*, and *mirror right* conditions. The shaded area represents standard error of the mean (SEM) computed separately for each condition. **(Bottom)** Topographic images for (A) *direct right*, (B) *mirror right*, (C) *direct left*, and (D) the difference between *mirror right* and *direct right* conditions.

The grand-averaged traces for the pooled data of healthy participants are presented in Figure 3, top for *direct left, direct right*, and *mirror right* conditions. Like the well-known readiness potentials, we observe an increase in negativity for all the three conditions with respect to the baseline period, however with spatial differences described below. (i) *Direct left* and *direct right* showed higher negative activity on the contralateral side, i.e., right (C2, CP2, FC4, C4, and CP4) and left-side electrodes (C1, CP1, FC3, C3, and CP3) compared to the ipsilateral electrodes, respectively. (ii) The contralateral negativity is highest for the *direct left* condition, with peak negative activity at the C2 electrode (mean ± SEM: −18.0 ± 1.0 μV at 0.4 s), negativity at the Cz electrode (−17.0 ± 1.1 μV at 0.4 s), and negativity at the C1 electrode (−14.5 ± 0.75 at 0.4 s). For the *direct right* condition, we observe similar higher negativity at the contralateral electrode (C1, −12 ± 1.2 μV at 0.2 s) compared to the ipsilateral electrode maximal negativity (C2, −10.0 ± 0.7 μV at 0.3 s), but with the highest negativity at the vertex electrode (Cz, −14 ± 0.8 μV at 0.25 s). (iii) Interestingly, the *mirror right* condition showed bilateral negativity at the central electrodes, such as C1 (−14.0 ± 0.8 at 0.25 s), Cz (−14.8 ± 0.8 at 0.30 s), and C2 (−10.0 ± 0.7 at 0.30 s).

We show the topographic maps of MRCPs obtained using averaged potentials in the time window of 775–800 ms in Figure 3 (bottom). The data were re-referenced to the vertex electrode (Cz) to highlight the topographic laterality of the negativity. The *direct right* condition displayed negativity at the left-side electrodes (at FC3, C3, C1, CP3, and CP1; Figure 3, bottom B), whereas *direct left* showed negativity at the contralateral fronto-central and central electrodes (at FC4, C2, C4, and CP2; Figure 3, bottom B) confirming the lateralization. Interestingly, the *mirror right* condition exhibited a slight bilateral activity with a higher negativity in the contralateral fronto-central and central electrodes (e.g., FC3, C1, and C3; Figure 3, bottom C), including a few ipsilateral electrodes.

The difference between *mirror right* and *direct right* in Figure 3 (bottom D) shows bilateral negativity (e.g., at FC3, FC4, and C2 electrodes).

We performed one-way ANOVA at the group level for the condition factor (three-leveled factor: direct right, direct left, and mirror right). The results revealed significant differences (*p* < 0.01) at electrodes FC3 (*F* = 7.98), FCz (*F* = 18.7), FC4 (*F* = 33.4), C3 (*F* = 12.6), Cz (*F* = 21.6), C2 (*F* = 29.9), C4 (*F* = 41.7), CPz (*F* = 31.5), CP2 (*F* = 31.3), and CP4 (*F* = 32.4). Pairwise tests between *direct right* and *direct left* revealed significant differences (*p* < 0.01) at fronto-central, central, and centro-parietal electrodes [99.9% CIs (4.11, 17.2) at FCz, (8.11, 20.8) at FC4, (4.78, 17.1) at Cz, (6.26, 17.2) at C2, (8.89, 23) at C4, (6.65, 18.7) at CPz, (4.68, 15.5) at CP2, and (6.21, 17.3) at CP4]. Similarly, a significant difference between direct right and mirror right was observed at the FC4 electrode [99.9%, CI (0.81, 13)].

### Stroke Participant

**Figure 4:**
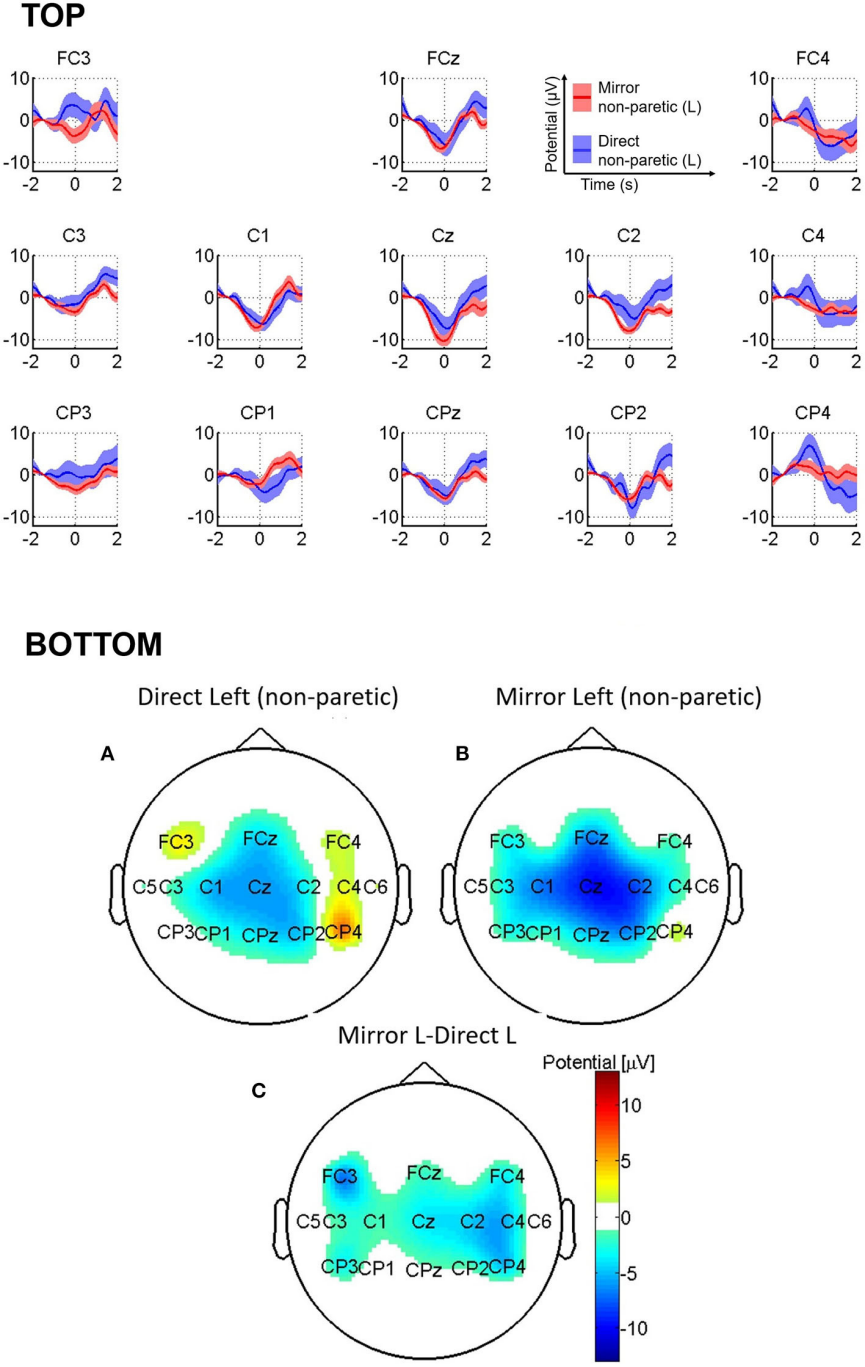
**(Top)** Grand-average MRCPs of the stroke participant for *direct left* (non-paretic) and *mirror left* (non-paretic). The shaded area represents standard error of the mean (SEM) computed separately. **(Bottom)** Topographic image of stroke participant's average MRCPs in the window (−225 to –200) ms for (A) *direct left (non-paretic)* and (B) *mirror left (non-paretic)*, and (C) the difference between *mirror left (non-paretic)* and *direct left (non-paretic)* conditions.

We present the grand-average MRCPs of the stroke participant in Figure 4, top for *direct left (non-paretic)* and *mirror left (non-paretic)* conditions. In the *direct left (non-paretic)* condition, we observed negative potentials at the central electrodes, but with less clear lateralization in the maximal negative activity (C1, −6.2 ± 1.5 μV at 0.15 s; Cz, −7.3 ± 3.0 μV at 0.14 s; and C2, −5.0 ± 3.0 μV at 0.25 s). Interestingly, the *mirror left (non-paretic)* condition elicited similar patterns yet with higher negativity (C1, −7.1 ± 1.0 μV, at 0.14 s; Cz, −10.4 ± 1.2 μV, at 0.15 s; and C2, −7.8 ± 0.8 μV, at 0.05 s). Overall, the appearance of the negative peak was earlier than that of the healthy participants.

We show topographic maps of MRCP activity in Figure 4 (bottom), with MRCP data averaged between −225 to −200 ms. Tukey's test resulted in a significant difference between *mirror left (non-paretic)* and *direct left (non-paretic)* conditions on both the ipsilesional {FC3 electrode [95% CI (0.758, 13.2)] and contralesional sides [C2 electrode 95% CI (0.0414, 9.52)].

### Outcome Measures

The NIHSS score decreased from six at pre-intervention (T1) to three at discharge (T2), corresponding to a mild impairment (Table 1). The score further reduced to 2 at follow-up (T3). The FAT score did not change from T1 to T2 (=3 points) but improved at T3, to 5 points. We observed clinically important improvement (+6 points; with main contributions from the synergies of shoulder, elbow, and forearm) in FMA-UE at T2 compared to T1 (+6 points) that further improved to 54 points at T3.

**Table 1:** Assessments from the stroke participant [NIHSS, FAT, and FMA-UE at pre- (T1) and post-intervention (T2) and at follow-up (T3)].

**Assessment**	**Pre (T1)**	**Post (T2)**	**Follow-up (T3)**
NIHSS (max, 42)	6	3	2
FAT (max, 5)	3	3	5
FMA-UE			
A.Upper extremity synergies (max, 36)	18	24	29
B. Wrist (max, 10)	0	2	7
C. Hand (max, 14)	3	2	13
D. Coordination/speed (max, 6)	4	5	5
Motor function total A–D (max, 66)	26	32	54
Sensation (max, 12)	8	8	12
Passive joint motion (max, 24)	22	21	21
Joint pain (max, 24)	21	24	22

*NIHSS, National Institutes of Health Stroke Scale; FAT, Frenchay Arm Test; FMA-UE, Fugl–Meyer Motor Assessment—upper extremity*.

## Discussion

The present study explored the nature of MRCPs due to MVF delivered using an embodied visual feedback on a computer screen during reach movements in a stroke participant and age-matched adults. In healthy participants, we reported MRCPs with peak activities in the hemisphere contralateral to the active arm in direct left and direct right situations, as expected (9, 15). Interestingly, the direct left condition resulted in stronger negative potentials at the contralateral electrodes compared to direct right. It is perhaps due to non-dominant hand movements in VR feedback being more demanding than those of the dominant arm (22). During VR-MVF, we observed the strongest negativity at the central electrodes, contralateral to the active arm, likely because performing the planar reach movement is even more complex in mirror than in direct feedback. Interestingly, we observed statistically different negativity at the ipsilateral fronto-central electrode, FC4, in the mirror condition compared to the direct condition, which supports our hypothesis of bilateral cortical excitability modulation with VR-MVF.

Contrary to the healthy participants, for the stroke participant, the direct left (non-paretic) condition showed less lateralization with similar negativity patterns at C1 and Cz electrodes. The recruited patient presented a pontine lesion, i.e., the anatomical pathways mediating interhemispheric interactions were preserved, a condition not always possible in patients with stroke. Thus, this difference could be due to natural individual differences or being stroke specific, which should be confirmed with more data of different stroke profiles in different phases of the recovery. We also observed a reduced latency of the negative peak compared to healthy participants, which has previously been reported in the literature (14). In the *mirror left* condition, we found stronger and bilateral (symmetric) activity at the central electrodes, which is in line with the healthy participant data. This is likely due to the interhemispheric communication through *mirror* feedback (2), which may be intact to some degree.

It is worth noting that over the three intervention days (days 9–11), the patient performed 185 *direct right* (using the paretic arm), 210 *direct left* (using the non-paretic arm), and 215 *mirror left* (using the non-paretic arm) repetitions, which represents high dosage during the acute hospitalization period. Pre- and post-VR task motor assessment showed an increase in the FMA-UE score of 6 points, which is more than the minimal clinically important difference in FMA-UE score (17). At follow-up, the score increased by 22 points along with a 4-point improvement in sensation. When answering a self-reported questionnaire, the stroke participant also announced high levels of concentration, enjoyment, and relaxation, although there is an increased level of fatigue while performing the VR exercises. The results of a review of over 34 studies on the effect of VR therapy after stroke by Ahn and Hwang (23) showed that VR approaches are effective in improving upper extremity function as well as independent activities in stroke survivors. Notably, Islam and Brunner (24) reported that the extra cost of VR therapy was outweighed by the reduced therapist supervision time, the increased patient motivation, and the expected decreasing VR system costs in the nearer future.

Calabrò et al. (25) showed that robotic-based rehabilitation combined with VR in patients with chronic hemiparesis induced an improvement in gait and balance. EEG data suggest that the use of VR may affect several brain areas (probably encompassing the mirror neuron system) involved in motor planning and learning, thus leading to an enhanced motor performance. Furthermore, Weber et al. (26) also integrated the well-known mirror therapy into an immersive virtual reality. However, our study is the only study to correlate the benefit of outcome with neurophysiological evaluation.

## Conclusion

In the current proof-of-concept study, we report that cortical excitability is likely modulated using VR-MVF in both healthy participants and a stroke patient and hypothesize that such an intervention may potentially activate neural mechanisms involved in stroke recovery.

## Data Availability Statement

The original contributions presented in the study are included in the article/supplementary material. Further inquiries can be directed to the corresponding author/s.

## Ethics Statement

The studies involving human participants were reviewed and approved by The Human Research Ethics Committee of the Canton of Vaud, Switzerland (CER-VD). The patients/participants provided their written informed consent to participate in this study.

## Author Contributions

GG and DP-M contributed to project design and its conception and writing the first draft of the manuscript. TR performed data analysis. JJ contributed to the practical execution of the study. KD was the principal investigator of the study and contributed to planning and overseeing the study. All authors contributed to the manuscript revision, read, and approved the submitted version.

## Conflict of Interest

GG and DP-M were employees at MindMaze SA, Lausanne, Switzerland at the time of the study. The remaining authors declare that the research was conducted in the absence of any commercial or financial relationships that could be construed as a potential conflict of interest.
